# Identification of major QTLs for seed vigor and growth-related traits using a biparental population in soybean

**DOI:** 10.3389/fgene.2025.1695593

**Published:** 2026-01-14

**Authors:** Neeraj Kumar, James R. Smith, Jeffery D. Ray, Jason D. Gillman, Nacer Bellaloui

**Affiliations:** 1 USDA, Agriculture Research Service, Crop Genetics Research Unit, Stoneville, MS, United States; 2 Plant Genetics Research Unit USDA-ARS, 205 Curtis Hall, University of Missouri, Columbia, MO, United States

**Keywords:** growth-related traits, high-density genetic map, QTL mapping, seed vigor, soybean

## Abstract

Soybean [*Glycine max* (L.) Merr.] is one of the most widely cultivated crops globally and serves as a primary source of plant-based protein and oil for human consumption. Seed vigor is a critical trait for germination and rapid seedling establishment, especially under sub-optimal environmental conditions. Genetic control of seed vigor measured as accelerated aging (AA%) was investigated using a quantitative trait locus (QTL) mapping approach. Associations of AA with six other traits: pubescence color (PC), beginning bloom (R1), reproductive period (RP), maturity (R8), plant height (PH), and stem termination (ST) were examined. A recombinant inbred line (RIL) population (247 F_6_-derived RILs) from a cross between DS25-1 and DT97-4290 was developed and genotyped using genotyping-by-sequencing (GBS), which yielded a set of 8,445 curated single-nucleotide polymorphism (SNP) markers from ∼90,000 SNPs. A subset of 201 RILs was phenotyped in Stoneville, Mississippi, over 3 years (2017–2019). A molecular linkage map was constructed using the SNP marker dataset, and composite interval mapping was performed using the R/qtl package. In total, 33 QTLs associated with seven phenotypic traits were identified across 12 chromosomes, using means of individual environments and best linear unbiased prediction (BLUP). Phenotypic variation of individual QTLs ranged from 0.56% to 89.77%, and the additive effects varied from −10.52% to 15.12%. Twelve QTLs detected across multiple environments on Gm03, Gm06, Gm07, and Gm19 were classified as stable. Notably, four genomic regions demonstrated QTL co-localization: Gm06 (all traits except ST), Gm07 (AA, R1, RP, and R8), Gm03 (AA and R1), and Gm19 (PH, R8, and ST). Major QTLs were in proximity to previously known genes: the *T* locus for pubescence color (Gm06), *E1* (Gm06) and *E11* (Gm07) loci for flowering and maturity, and *Dt1* (Gm19) for stem termination. The closest SNPs associated with stable QTLs can be used to develop KASP markers for marker-assisted selection programs.

## Introduction

Soybean [*Glycine max* (L.) Merr.] is a major global crop; however, seed yields in farmers’ fields rarely approach the yield potential of modern soybean cultivars. Achieving high yields and superior seed quality relies on multiple factors, including selecting high-yielding varieties with strong seed vigor, high germination rates, and successful seedling establishment, and effective management practices. These practices also involve minimizing biotic and abiotic stress throughout the crop season.

Seed vigor reflects a seed’s capacity to quickly germinate and establish a stand in a wide range of environmental conditions. Poor seed emergence directly reduces crop yield by decreasing plant density per unit area due to low germination rates and weak vigor (Han et al., 2014; Börner et al., 2018). Seed vigor is governed by complex interactions between internal (endogenous) and external (exogenous) factors that influence seed development, harvest, and seed storage (Bentsink et al., 2000). The endogenous factors include chlorophyll content (as seen in chlorophyllous seeds), seed coat structure, phytohormone levels, and the integrity of proteins and nucleic acids (DNA and RNA), along with their repair mechanisms (Zhou et al., 2020). Exogenous factors begin to influence physiological maturity and continue to do so throughout seed storage. These include environmental conditions such as sub-optimal temperatures, relative humidity, and oxygen levels during seed storage (Nadarajan et al., 2023). High temperatures and humidity during seed storage can significantly reduce seed viability by disrupting critical biochemical processes. These adverse conditions enhance hydrolytic enzyme activity, increase respiration rates, and promote lipid peroxidation, thereby elevating free fatty acid levels (Copeland and McDonald, 2001; Wang et al., 2018).

In the mid-south of the United States, soybeans are grown using an early soybean production system (ESPS), where planting occurs early (April), and optimally, the crop is harvested early (August) as well. However, when harvest is delayed, the seed can be exposed to adverse weather conditions (Pinheiro et al., 2021), increasing the risk of seed deterioration from microbes that cause rot and prevent the seed from sprouting within the pod due to excess moisture. Eventually, both seed quality and composition are compromised. Such mature seed damage reduces seed vigor and can lead to substantial revenue losses when the grain is eventually marketed (Heatherly, 1999). Yet, the ESPS is used because it has been shown to provide consistent yield advantages and increased profitability under both irrigated and non-irrigated conditions (Heatherly and Spurlock, 1999). Gaining insight into the genetic basis of seed vigor can facilitate the development of cultivars with enhanced viability and seed quality.

Previously, the screening of genetically diverse exotic soybean germplasm, including 25 ancestral lines of U.S. cultivars, revealed poor germination of the ancestor lines under ESPS conditions (Smith et al., 2008; Smith et al., 2017; Gillman et al., 2020). The average germination rates for these ancestors were 50.8% in standard tests and 37.9% under accelerated aging (AA). Moreover, ancestral seeds exhibited a high incidence of hard seeds (13.2%), wrinkling (29.6%), and *Phomopsis longicolla* infection (22.5%), demonstrating that the use of the ancestor gene pool alone would likely be insufficient to improve seed vigor in modern cultivars. In contrast, seeds of an unadapted exotic landrace, PI 587982A, maintained 92% and 90% germination in standard and AA tests, respectively, with no hard, wrinkled, or diseased seeds (Smith et al., 2008). One of the parental lines used in this study, DS25-1 (PI 684675: https://npgsweb.ars-grin.gov/gringlobal/), was derived from PI 587982A and was crossed with DT97-4290 (Paris et al., 2006), a low-vigor line, to develop the RIL population for the current study.

In the last 2 decades, genomic approaches, such as genome-wide association studies (GWAS) and quantitative trait locus (QTL) mapping, have been successfully used to dissect various complex traits related to agronomy, growth period, growth habits, flowering, maturity, and tolerance to biotic and abiotic stresses (see review from Xia et al., 2013; Zhang et al., 2015; Liu et al., 2016; Zimmer et al., 2021; Kim et al., 2023; Liu et al., 2023). Fang et al. (2017) used GWAS to identify several candidate genes linked to various important traits related to growth period, architecture, seed yield, and nutrient composition using large collections of soybean germplasm (809 accessions). Previously, biparental RIL populations were widely used to map QTLs associated with complex quantitative traits, including seed yield, seed size, and seed composition (Yao et al., 2023; Gao et al., 2024).

QTL mapping remains a powerful tool for dissecting the genetic architecture of complex traits in crop species. In previous years, significant efforts were made to identify loci associated with high seed vigor and storability in various crops through genetic studies. In *Arabidopsis*, several loci associated with seed storability were identified (Bentsink et al., 2000; Clerkx et al., 2004). In rice, numerous QTL mapping studies using RIL populations pinpointed loci for early seed vigor (Yang et al., 2019; Zhang et al., 2017), maturity-related seed vigor (Liu et al., 2014; Lai et al., 2016), and seed longevity (Raquid et al., 2021). Integrative analyses, combining GWAS, QTL mapping, and transcriptomic data, have further identified candidate genes for seed vigor in rice (Yang et al., 2021). Similarly, studies in maize using RIL populations uncovered several QTLs related to seed maturity at harvest (Jing-bao et al., 2011), seed longevity (Han et al., 2018), tolerance to stressful germination conditions (Han et al., 2014), and artificial aging tolerance (Wu et al., 2019). In wheat, Shi et al. (2020) conducted QTL and candidate gene analysis to identify genes associated with seed vigor. Seed vigor studies have also been conducted in barley (Nagel et al., 2016; Thabet et al., 2018) and oat using QTL mapping and GWAS (Huang et al., 2020). To date, only a few studies have identified loci associated with seed vigor and storability in soybean using QTL mapping approaches. Dargahi et al. (2014) reported three loci controlling seed storability on linkage groups C1 (Gm04), F (Gm13), and L (Gm19). Zhang et al. (2019) identified 34 stable QTLs for seed storability across 11 chromosomes using two different RIL populations. Wang et al. (2021) identified two major QTLs and eight potential minor QTL on chromosomes 3, 6, 9, 11, 15, 16, 17, and 19 for traits related to accelerated aging using a biparental mapping population using seeds conserved at −20 °C. Jiamtae et al. (2022) reported five QTLs associated with seed viability and seed vigor on chromosomes 2, 6, and 8, which were co-localized with loci linked to seed variability, maturity, germination, hardness, and micromorphology.

For the current study, we constructed a high-density genetic linkage map to map QTLs for seed vigor and growth-related traits using an entire RIL population developed from a cross between two parental lines (DS25-1 and DT97-4290). The genetic map, in conjunction with phenotypic data collected over 3 years (2017–2019), was used for composite interval mapping (CIM) using the R/qtl package (Broman and Sen, 2009). Several consistent QTLs across the environments were identified for seven traits (AA, PC, R1, RP, R8, ST, and PH), including both novel and previously identified QTLs and candidate genes. In addition, we identified four genomic regions that harbor multiple stable QTLs for different traits. SNPs associated with stable QTLs for the corresponding traits can be converted into KASP markers to facilitate marker-assisted selection for improving seed vigor and overall soybean productivity in improved cultivars.

## Materials and methods

### Development of the RIL population

The cross was made between DS25-1 (Smith et al., 2017) and DT97-4290 (Paris et al., 2006) at Stoneville, MS, in 2012. DS25-1 is a maturity group (MG) IV line and is tolerant to heat-induced seed degradation. DT97-4290 is a high-yielding MG IV public germplasm line with moderate resistance to charcoal rot (*Macrophomina phaseolina* [Tassi] Goid.) but susceptibility to heat-induced seed degradation reduced germination (Chebrolu et al., 2016) and increased levels of seed wrinkling (Narayanan et al., 2020), purple seed stain, and mold (Smith et al., 2025). In 2013, F_2_ seeds were harvested at the USDA Tropical Agriculture Research Station at Isabela, Puerto Rico, and planted at Stoneville. In the fall, 247 individual F_2_ plants were randomly selected to initiate the RIL population. The F_3_ and F_5_ RIL generations were advanced by randomly selecting a single plant from each heterogeneous progeny row at Homestead, FL (27 Farms of Homestead, Inc.), during the winters of 2013/2014 and 2014/2015. In contrast, the F_4_ and F_6_ generations were advanced at Stoneville during the summers of 2014 and 2015 by the same method. Single F_6_-derived F_7_ seeds were harvested in 2015 for seed increase and further field evaluations.

### Field evaluation

All 247 RILs were planted on 10 April 2017 in 2.7 m-long single-row plots with a row spacing of 0.91 m with two replications using a randomized complete block design (RCBD) at Stoneville at the Delta Research and Extension Center (33° 26′ N, −90° 54′ W) in 2017. Seeds were sown with a machine planter into rows at a depth of 2.5 cm, with a seeding rate of 25 seeds m^−1^ of row. The soil type was a Sharkey clay soil (very fine, smectitic, thermic Chromic Epiaquert). The 2017 experiment was furrow-irrigated as needed to avoid water-deficit stress and ensure adequate seed production for future years of testing. After planting and emergence, Reflex (Syngenta, DE, United States) and Select (Valent Corporation, CA, United States) herbicides, along with multiple timely cultivations, were used for weed control. Non-irrigated (rainfed) Stoneville plantings were made on 20 April 2018 and 22 April 2019 using a subset of 201 RILs, with lines maturing earlier than Pella 86 (Fehr et al., 1987) and later than Dillon (Shipe et al., 1997) not planted in the second and third years. The same experimental procedures as noted above, except for irrigation, were used in 2018 and 2019. The checks used in 2017 were early MG III Pella 86, late MG III Maverick (Sleper et al., 1998), late MG IV parents DT97-4290 and DS25-1, MG V Osage (Chen et al., 2007), and MG VI Dillon. In 2018 and 2019, the same checks were used with the addition of the following: mid MG III 7037–12111 (an unreleased high germinability line derived from PI 587982A × N98-4445A), late MG III germplasm lines DS65-1 and LG03-4561-14, early MG IV DS34-1 (which is referred to as 34-3-1-2-4-1), early MG IV commercial cultivars Progeny 4211 and AG4232, and late MG IV and mid MG V commercial cultivars C4926 and AG5606 (https://legacy.soybase.org/uniformtrial/index.php?page=lines&filter).

### Trait phenotyping

Growth stages (Fehr et al., 1977) R1 (beginning bloom) and R8 (full maturity) were recorded in days after planting (DAP) for each plot. In each year, R1 was estimated weekly when 50% of the plants in a plot had flowered, whereas R8 was estimated twice weekly in each plot when 95% of the pods had reached their mature pod color. The duration of the reproductive period (RP) was estimated in days as the difference between R1 and R8. Pubescence color (PC) and stem termination (ST) type were recorded for each plot at R8. The RIL population was segregating for two pubescence colors, as one parent (DS25-1) had gray pubescence, whereas the other parent (DT97-4290) had tawny pubescence. Similarly, the population segregated for stem termination habit, as one parent (DS25-1) was a determinate (D) type, whereas the other parent (DT97-4290) was an indeterminate (I) type. Plant height (PH) for each plot was estimated in 2019, measured as the distance in cm from the ground to the tip of the plant. PH estimates were taken on three plants per plot and then averaged. The timely harvest of seed was completed for each plot shortly after full maturity by hand-harvesting each plot, followed by storing/drying the cut plants at 21 °C and 60% relative humidity (RH). Harvested plants were threshed using an Almaco bundle thresher (Almaco, Nevada, IA). Split seeds and debris (sticks, dirt, etc.) were removed from each seed lot at the time of threshing, whereas damaged seed (wrinkled, shriveled, discolored, green, purple, moldy, insect feeding) was retained for later grading. The seed of each line was stored at 21 °C and 60% RH until all lines were harvested, threshed, and cleaned of debris. Accelerated aging (%; AA) tests were conducted in 2018 and 2019 at the State Seed Testing Laboratory (Mississippi State, MS) following standard protocols of the Association of Official Seed Analysts (2002). For each seed lot, ∼42 g of random seed was sampled, and 200 seeds were divided into four replicates of 50. Damaged or abnormal seeds were removed, and seed moisture content was adjusted to 10%–12%. Each replicate was placed as a single layer on a screen tray in an accelerated-aging box containing ∼40 mL distilled water to maintain nearly 100% relative humidity. Boxes were sealed and incubated at 41 °C–45 °C for 72 h to simulate rapid aging. After aging, boxes were cooled for 1 h before opening to prevent moisture loss. Seeds were then tested for germination on moist paper or in moist sand using the rolled towel or between-paper method at 25 °C ± 2 °C for 7 days. The percentage of normal seedlings, determined by AOSA criteria, was recorded as the accelerated aging value.

$$AA\left(\%\right)=\frac{\text{Number}\;\text{of}\;\text{normal}\;\text{seedlings}\;\text{after}\;\text{aging}}{\text{Total}\;\text{number}\;\text{of}\;\text{seeds}\;\text{tested}}\times 100.$$

### Statistical analyses

To study the relationship among the phenotypic traits, a Pearson’s correlation coefficient matrix was generated using *corr_plot* and *plot.corr_coef* functions of the *metan* package in R software (R Foundation, 2024). The variance components for genotypes (i.e., RILs) were estimated for quantitative traits (AA, R1, RP, R8, and PH) using the *lme4()* function in R (Bates et al., 2015). We used *y*_*ijkl*_ *= µ + E*_*j*_ *+ G*_*j*_ *+* (*GE*)_*ij*_ *+ R*_*k(j)*_ *+ ε*_*ijkl*_, where environment (year) was treated as a fixed effect, and genotype (*G*_*i*_), genotype × environment interaction (*GE*)_*ij*_, and replication within year *R*_*k(j)*_ were fitted as random effects. The mixed linear models were optimized using restricted maximum likelihood and were used to determine variance components for each random effect. Best linear unbiased predictions (BLUPs) were also calculated for the above traits using the random effect of each genotype within and across environments using the *lme4* package in R software (Bates et al., 2015). Broad-sense heritability was calculated on an entry-mean basis according to Holland et al. (2003), with the variance components estimated using a model where all variables were treated as random.

$$H^{2}=\frac{\sigma_{G}^{2}}{\sigma_{G}^{2}+\frac{\sigma_{G\times R}^{2}}{R}+\frac{\sigma_{G\times Y}^{2}}{Y}+\frac{\sigma_{E}^{2}}{RY}},$$

where G is genotype, R is replicate, Y is year, and E is error.

### Production of genotyping-by-sequencing (GBS) markers and data processing

Leaf samples from each of the 247 RILs and both parental lines were collected from experimental field plots grown at Stoneville, MS, in 2015. The collected leaf sample was lyophilized using a Model 2400 freeze dryer (The Freeze Dry Company, Nisswa, MN 56468, United States). Genomic DNA was extracted employing a DNA isolation kit (Promega, Madison, WI 53711, United States) in a Maxwell 16™ automated DNA isolation machine. The genotyping was carried out using GBS markers technology (Elshire et al., 2011) by LGC Genomics GmbH (Berlin, Germany) following their established protocols (https://www.biosearchtech.com/) for normalized GBS (nGBS) with MsII and Illumina NextSeq (2 × 150 bp) sequencing. DNA sequences were processed by LGC Genomics bioinformaticists for quality, and the GBS tags were aligned to the “Williams 82” soybean reference genome assembly 1 (https://www.soybase.org/). The aligned sequences were separated for each plant sample using the in-line barcode sequences, and then the reads were processed to remove the barcode and adapter sequences. For sequence alignment, the Burrows–Wheeler Aligner (BWA v.0.7.12; http://bio-bwa.sourceforge.net/) program was used (Li and Durbin, 2009), and SNPs were called using Freebayes v.1.92-16 (Garrison and Marth, 2012).

### Quality control of GBS-SNP markers

Prior to genetic mapping, monomorphic SNPs that had excessive heterozygous or missing values (>15%) or did not follow a 1:1 segregation ratio (chi-square P value ≤0.01) were removed. Missing data for SNPs with <15% missing data were imputed using an LD-kNNi method, which is based on a k-nearest-neighbor genotype (Money et al., 2015). This analysis was implemented using TASSEL software (https://www.maizegenetics.net/tassel). This resulted in a set of 8,925 (8,890 + 35 scaffold) high-quality-filtered SNPs, of which 8,890 polymorphic SNPs were used to construct a genetic linkage map.

### Construction of the genetic linkage map

Prior to linkage map construction, the genotypes of all 247 RILs were converted into ABH allele format, where allele “A” is from DS25-1, “B” is from DT97-4290, and “H” is heterozygous. All polymorphic SNPs (8,890) were used for the construction of the linkage map using R/qtl (Broman et al., 2003). The ABH data format was used to form linkage groups with a logarithm of odds (LOD) threshold ≥3.0 and a maximum recombination frequency of 0.35. In total, 8,445 SNPs were assigned to 22 linkage groups (LGs), corresponding to 20 soybean chromosomes. The LGs were assigned to different chromosomes based on the genomic position of SNPs identified during SNP calling. The markers within the LGs were ordered using the “ordermarkers” and “ripple” functions in R/qtl to minimize the length of linkage maps. The genetic distance between the markers was calculated in centiMorgans (cM) using the Kosambi mapping function (Kosambi, 1944).

### QTL analysis and candidate gene identification

QTL analysis was performed using the genetic linkage map described above, along with phenotypic data from seven traits (AA, PC, R1, RP, R8, PH, and ST). Mean values from individual environments of each phenotypic trait were used for QTL analysis. The phenotypic observations recorded for discrete traits (PC and ST) were converted into a numerical variable (binary coding) prior to data analysis. For PC, gray pubescence color was coded as “0,” tawny as “2,” and a heterozygous individual was coded as “1.” For the stem termination habit, the indeterminate type was coded as “0,” the determinate type as “2,” and a heterozygous individual was recorded as “1.” The individual environment includes Stoneville 2017 (I), Stoneville 2018 (II), and Stoneville 2019 (III). Across-environment QTL analysis was carried out using BLUP values for the continuous traits (AA, R1, RP, R8, and PH), which were denoted as IV. We performed composite interval mapping (CIM) via the “cim” function for QTL detection, following the methodology outlined in *R/qtl* (Broman and Sen, 2009). CIM analysis was performed using multiple QTL regression models using R software (Broman and Sen, 2009). A minimum logarithm of odds (LOD) score ≥2.5 was used to determine the presence of a putative QTL, whereas the threshold LOD scores were computed using 1,000 permutations. A QTL detected in at least 50% or more environments was classified as a stable QTL (Cao et al., 2019; Bazzer et al., 2020). Additionally, a QTL was classified as “Major” if it appeared with a LOD score ≥4.0 and phenotypic variation explained (PVE) ≥ 10%. Candidate genes were identified in SoyBase (https://www.soybase.org/) using the Williams 82.a1 (wm82.a1) reference genome (Schmutz et al., 2010). The intervals of stable QTLs were used to search for the candidate genes of each trait. Identified candidate genes were analyzed using Gene Ontology (GO) to reveal their molecular function via SoyBase (https://www.soybase.org/). For the corresponding gene positions from the Glyma.wm82.a1 assembly to Glyma.wm82.a4, we used “gene model correspondence lookup” available at SoyBase (https://www.soybase.org/).

## Results

### Phenotypic diversity between parents and the RIL population

**FIGURE 1:**
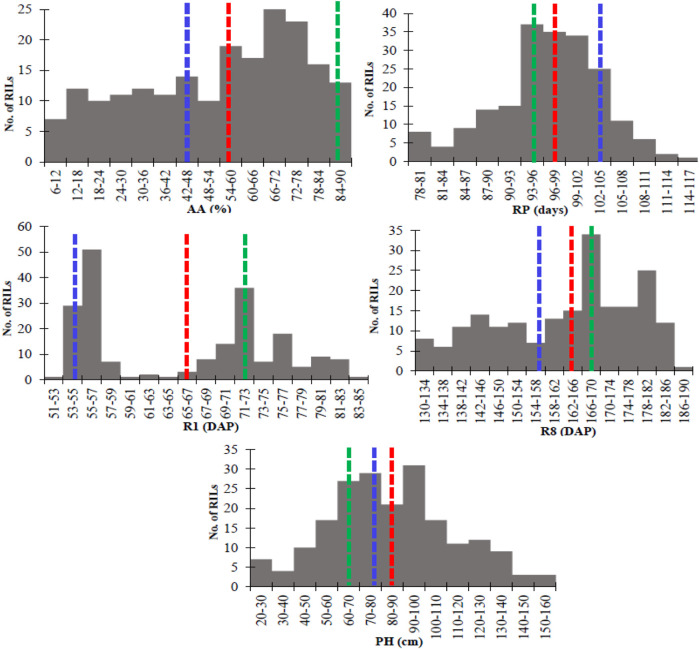
Frequency distribution of the means of different phenotypic traits of the recombinant inbred lines (RILs) derived from DS25-1 and DT97-4290. AA, accelerated aging (%); R1, beginning bloom; RP, reproductive period; R8, maturity; PH, plant height. Dashed green, blue, and red lines represent the means of the DS25-1, DT97-4290, and RIL populations, respectively. Means values of each trait were used for R1, RP, and R8 (2017–2019), AA (2018–2019), and PH (2019).

The parental lines (DS25-1 and DT97-4290) and the RIL population exhibited a wide range of phenotypic diversity across the quantitative traits (Table 1). DS25-1 displayed a significantly higher seed vigor score (accelerated aging, AA), at 85%, than DT97-4290, which had a score of 45% across years (2018–2019). DT97-4290 was an early-blooming line, reaching the R1 stage (beginning bloom) at 55 DAP, while DS25-1 reached R1, 17 days later, at 72 DAP. Similarly, DT97-4290 reached full maturity (R8) at 158 DAP, which was 10 days earlier than DS25-1 (168 DAP). However, the reproductive period (RP) was shorter in DS25-1 (96 days) than in DT97-4290 (103 days). In terms of plant height (PH), DT97-4290 (81 cm) was significantly taller than DS25-1 (66 cm). The RIL population showed a broad range of variation across all traits (Table 1), with transgressive segregation observed for all quantitative traits (AA, R1, RP, R8, and PH). The AA of the RILs ranged from 6% to 89%, with an average of 54%, which was significantly less than that of the parents. The RILs’ PH ranged from 23 cm to 157 cm, with an average (84 cm) that was greater than the parental means. However, the means for three traits (R1, RP, and R8) in the RIL population were very close to the parental means. Variance component analysis showed that genotypic variance in the RILs, genotype × environment (G × E), replication, and year had significant contributions to total phenotypic variance for each trait (AA, R1, RP, and R8) in this population (Supplementary Table S1). The environmental effects on phenotypic trait values were primarily attributed to year and replication for R1, RP, and R8, while AA showed a highly significant genotype × year interaction. The RIL population exhibited continuous variation in a near-normal distribution for RP and PH (Figure 1), while AA showed a slight negative skew. The distribution for R1 and R8 was bimodal.

**TABLE 1:** Descriptive statistics of the parents (DS25-1 and DT97-4290) and RIL population for plant height (PH) for a single year (2019), for accelerated aging (AA%) for 2 years (2018–2019), and beginning bloom (R1), reproductive period (RP), and maturity (R8) for 3 years (2017–2019).

Trait^a^	Parental lines of RILs		RIL population		
	DS25-1	DT97-4290	Mean	Range	CV
AA	85	45	54 (1.6)	6–89	31
R1 (DAP)	72	55	66 (0.7)	53–84	14
RP (days)	95	103	97 (0.5)	79–116	7
R8 (DAP)	168	158	163 (1.0)	130–187	9
PH (cm)	65	81	85 (1.9)	23–157	33

^a^ CV, coefficient of variation; DAP, days after planting. Value in parentheses is standard error.

### Heritability and the relationship between phenotypic traits

The broad-sense heritability was highest for R8 (0.96) and lowest for PH (0.52). Heritability was also high for R1 (0.88), RP (0.81), and AA (0.81) (Table 2). Pearson’s correlation coefficients among seven phenotypic traits are presented based on the means of phenotypic traits, including data for PH (2019), AA (2018–2019), and across the years for R1, RP, and R8 (2017–2019; Table 2). As expected, R1 and R8 showed strong, positive correlations across the 3 years (*r* = 0.87). However, R1 exhibited a moderate (*r* = 0.39) correlation with RP. Seed vigor (AA) exhibited moderate to strong positive correlations with R1, R8, and PH, but surprisingly, it had only a weak but significant (0.12, P ≤ 0.05) relationship with RP. PH showed a moderate to strong positive relationship with R1, RP, and R8 (*r* = 0.41–0.55). Stem termination (ST) exhibited a moderate positive relationship with PH (*r* = 0.38), whereas the remaining traits did not show any relationship with RP or showed weak negative correlations with ST (Table 2). In contrast, pubescence color (PC) showed a significant moderate-to-strong negative relationship with all other phenotypic traits (Table 2) except ST, where it showed a significant weak but positive correlation (*r* = 0.19).

**TABLE 2:** Estimates of trait heritabilities and Pearson’s correlation coefficients among phenotypic traits of the RIL population derived from a cross between DS25-1 and DT97-4290 across years.

Trait	PC	ST	R1	RP	R8	AA	H^2^
ST	0.19^**^	​	​	​	​	​	-
R1	−0.87^***^	−0.16^**^	​	​	​	​	0.88
RP	−0.38^***^	0.02	0.39^***^	​	​	​	0.81
R8	−0.78^***^	−0.1	0.87^***^	0.80^***^	​	​	0.96
AA	−0.62^***^	−0.04	0.66^***^	0.12^*^	0.49^***^	​	0.81
PH	−0.49^***^	0.38^***^	0.51^***^	0.41^***^	0.55^***^	0.42^***^	0.52

Data used for pubescence color (PC), stem termination (ST), and plant height (PH), for only a single year, for accelerated aging (AA) for 2 years (2018–2019), and for beginning bloom (R1), reproductive period (RP), and maturity (R8), across the 3 years (2017–2019).

H^2^, broad-sense heritability.

* , **, and *** indicate significance at the 0.05, 0.01, and 0.001 levels, respectively.

### Construction of the genetic linkage map

A genetic linkage map was constructed using 8,445 quality-filtered SNPs derived from GBS across 247 RILs. In total, 22 linkage groups were formed, with one linkage group assigned to each of the 20 soybean chromosomes, except for Gm09 and Gm12, which each formed two separate linkage groups (Table 3). The cumulative length of the genetic linkage map was 2,519.91 cM with an average marker distance of 0.43 cM. The full genetic map is shown in Supplementary Figure S1. The maximum marker density was observed on Gm04 (1 marker/0.09 cM), while the minimum marker density was found on Gm12 (1 marker/0.97 cM). Among the chromosomes, Gm18 had the highest number of markers (999 SNPs), while Gm12 harbored the fewest (137 SNPs).

**TABLE 3:** Summary of genetic linkage maps using the biparental RIL mapping population derived from a cross between DS25-1 and DT97-4290.

Linkage groups^a^	Mapped SNPs	LG length (cM)	Average distance between SNPs (cM)	Max spacing between SNPs (cM)
Gm01/LG_D1a	393	126.56	0.32	9.40
Gm02/LG_D1b	292	177.08	0.61	35.45
Gm03/LG_N	345	148.72	0.43	26.18
Gm04/LG_C1	714	61.29	0.09	20.05
Gm05/LG_A1	179	132.74	0.75	16.70
Gm06/LG_C2	382	170.13	0.45	19.81
Gm07/LG_M	236	111.8	0.48	13.75
Gm08/LG_A2	188	124.58	0.67	21.3
*Gm09/LG_K.1*	597	55.88	0.09	13.75
*Gm09/LG_K.2*	56	34.65	0.63	11.96
Gm10/LG_O	173	60.66	0.35	20.77
Gm11/LG_B1	139	106.14	0.77	29.59
*Gm12/LG_H.1*	57	54.35	0.97	12.62
*Gm12/LG_H.2*	80	45.44	0.6	14.60
Gm13/LG_F	570	185.85	0.33	23.79
Gm14/LG_B2	797	128.16	0.16	8.62
Gm15/LG_E	257	133.53	0.52	24.33
Gm16/LG_J	573	128.14	0.22	35.44
Gm17/LG_D2	484	141.18	0.29	26.55
Gm18/LG_G	999	145.41	0.14	11.44
Gm19/LG_L	589	126.74	0.22	23.19
Gm20/LG_I	345	120.89	0.35	9.01
​	8,445	2519.91	0.43	35.45

^a^cM, centiMorgan. Linkage groups (LGs) are italicized for chromosomes 09 and 12, where two LGs were made.

### QTL for PC, PH, ST, and AA

To validate the RIL population, pubescence color (PC), a well-characterized qualitative trait in soybeans, was used for QTL mapping. Our results confirmed the presence of a major QTL at ∼18.7 Mb on Gm06, which exhibited a very high LOD score (110.3) and phenotypic variation (89.77%) and had minimal additive effects (−0.75) (Supplementary Figure S2A). Additionally, a minor QTL was also identified on Gm05 (∼41.7 Mb), with a LOD score of 2.80 and accounting for <1% of the phenotypic variation (Table 4).

**TABLE 4:** QTL detected for pubescence color (PC), plant height (PH), and stem termination (ST) from the means of two replications of an individual environment, whereas for accelerated aging (AA%), 2 years of means (2018–2019) and estimated BLUPs were used for composite interval mapping using RIL population derived from DS25-1 and DT97-4290.

Trait^a^	QTL name	Chr/LG	QPP (Mb)	QPP (bp)	QGP (cM)	LOD	PVE (%)	AD effects	Env.	Parent allele
PC	qPC.1	Gm05/LG_A1	41.7	41,675,693	131.7	2.8	0.43	0.06	II	P1
	qPC.2	Gm06/LG_C2	18.7	18,666,488	117.1	110.3	89.77	0.90	II	P1
AA	qAA.1	Gm03/LG_N	29.1	29,076,783	56.2	2.8	3.05	−4.57	III	P2
	qAA.2	Gm03/LG_N	37.4	37,433,048	98.0	2.8	3.64	−3.16	IV	P2
	qAA.3	Gm05/LG_A1	41.7	41,675,693	131.7	3.1	3.32	4.81	III	P1
	​	Gm05/LG_A1	41.7	41,675,693	131.7	2.7	3.02	2.94	IV	P1
	qAA.4	Gm06/LG_C2	19.6	19,595,738	120.5	11.5	21.60	11.73	II	P1
	​	Gm06/LG_C2	19.6	19,616,246	120.5	24.6	32.63	14.91	III	P1
	​	Gm06/LG_C2	19.6	19,595,695	120.5	22.2	36.45	9.76	IV	P1
	qAA.5	Gm11/LG_B1	4.9	4,908,170	34.4	2.7	4.11	5.22	II	P1
	qAA.6	Gm11/LG_B1	7.2	7,224,445	48.2	2.6	2.70	4.30	III	P1
	qAA.7	Gm14/LG_B2	3.7	3,745,833	18.1	3.0	3.35	−4.69	III	P2
	qAA.8	Gm13/LG_F	27.9	27,940,207	68.3	4.4	5.64	4.03	IV	P1
PH	qPH.1	Gm06/LG_C2	20.3	20,291,873	121.5	16.2	28.49	15.52	III	P1
	qPH.2	Gm19/LG_L	45.8	45,757,122	90.9	7.2	12.12	−10.09	III	P2
ST	qST.1	Gm19/LG_L	45.8	45,757,122	90.9	27.8	65.63	−0.75	II	P2

^a^ Chr/LG, chromosome/linkage group; QPP, QTL, physical position (megabase); QGP, QTL, genetic position (centiMorgan); LOD, logarithm of the odds; PVE, phenotypic variation explained; AD, additive effects; Env., environment; I, Stoneville 2017; II, Stoneville 2018; III, Stoneville 2019; IV, BLUP, dataset; P1, DS25-1, P2, DT97-4290.

We identified a major QTL (*qST.1*) for stem termination (ST), with a high LOD score (27.8), on Gm19 (∼45.8 Mb). This QTL accounted for 65.63% of the phenotypic variation (Table 4; Supplementary Figure S2B) and had minimal additive effects (−0.75). For plant height (PH), two significant QTLs, qPH.1 and qPH.2, were identified on chromosomes Gm06 (20.3 Mb) and Gm19 (45.8 Mb), with LOD scores of 16.2 and 7.2, respectively (Supplementary Figure S2C). A major QTL (qPH.1) for PH accounted for 28.49% of the phenotypic variation, and another locus (qPH.2) accounted for 12.12% of the phenotypic variation. Additive effects were 15.52 and −10.09 (Table 4). Interestingly, QTLs for ST (*qST.1*) and PH (qPH.2) showed a large overlapping peak on Gm19 (∼45.7 Mb), with a common closest SNP (S19_45757122).

In total, eight significant QTLs for AA were identified (Table 4), distributed on six different chromosomes (Gm03, Gm05, Gm06, Gm11, Gm13, and Gm14). A major AA QTL (qAA.4) was identified with a LOD score of 24.6, which explained the largest proportion of phenotypic variation (32.63%) (Supplementary Figure S2D). It was also identified in two environments, suggesting greater stability. The other QTLs associated with AA are minor, with LOD scores ranging from 2.6 to 4.4 and accounting for phenotypic variation ranging from 2.70% to 5.64%. Of these eight QTLs, a locus (qAA.3) detected in two environments (III and IV) on Gm05, and another locus (qAA.4) detected on Gm06 across the environments (II, III, and IV), were both considered stable QTLs (Supplementary Table S2). The remaining QTLs detected on Gm03, Gm11, and Gm14 were significant only in single environments (Table 4).

### QTL for reproductive traits (R1, RP, and R8)

For R1, six QTLs (qR1.1 to qR1.6) were identified on five chromosomes (Gm01, Gm03, Gm06, Gm07, and Gm15), and the LOD scores ranged from 2.5 to 88.7 (Table 5; Supplementary Figure S2E). A major QTL identified on chromosome 6 (qR1.3) had the highest LOD score (88.7) and accounted for most of the phenotypic variation (82.58%). Comparatively, the other five QTLs were minor, with LOD scores ranging from 2.5 to 7.5 that explained a small portion of the phenotypic variation (0.83%–3.53%). Of these QTLs, three (qR1.3, qR1.5, and qR1.2) were consistently detected on Gm06 (∼18.0–20.3 Mb), Gm07 (3.9 Mb), and Gm03 (37.4–38.9 Mb) in a minimum of two to four environments (I, II, III, and IV) and had above-the-threshold LOD scores. They were considered stable QTLs. However, two additional significant QTLs were detected only in a single environment, each on Gm07 (7.5 Mb) and Gm15 (∼11.3 Mb).

**TABLE 5:** QTL detected for beginning bloom (R1), reproductive period (RP), and maturity (R8), from the means of each year (2017–2019) and the BLUP dataset following composite interval mapping using the RIL population derived from DS25-1 and DT97-4290.

Trait^a^	QTL name	Chr/LG	QPP (Mb)	QPP (bp)	QGP (cM)	LOD	PVE (%)	AD. effects	Env.	Parent allele
R1	qR1.1	Gm01/LG_D1a	1.3	1,335,864	6.7	2.5	1.01	0.94	IV	P1
	qR1.2	Gm03/LG_N	38.9	38,913,468	106.1	3.6	1.93	−1.22	II	P2
	​	Gm03/LG_N	39.3	39,272,951	107.1	2.7	0.57	0.91	IV	P2
	qR1.3	Gm06/LG_C2	19.0	18,959,143	117.8	48.7	66.83	7.17	II	P1
	​	Gm06/LG_C2	19.9	19,864,606	121.1	62.2	75.53	9.63	III	P1
	​	Gm06/LG_C2	20.3	20,291,912	121.5	88.7	82.58	10.57	I	P1
	​	Gm06/LG_C2	20.3	20,291,912	121.5	78.2	82.34	8.95	IV	P1
	qR1.4	Gm07/LG_M	4.0	3,978,484	18.8	7.5	3.53	−1.59	II	P2
	​	Gm07/LG_M	4.0	3,978,484	18.8	4.9	3.41	−2.02	III	P2
	​	Gm07/LG_M	4.0	3,978,484	18.8	4.9	2.24	−1.42	IV	P2
	qR1.5	Gm07/LG_M	7.5	7,540,731	40.5	5.2	0.83	−1.21	I	P2
	qR1.6	Gm15/LG_E	11.3	11,278,361	70.5	2.5	1.30	−1.31	III	P2
RP	qRP.1	Gm01/LG_D1a	56.3	56,264,066	126.3	2.6	3.03	−1.96	I	P2
	​	Gm01/LG_D1a	56.3	56,264,066	126.3	2.6	2.74	−1.28	IV	P2
	qRP.2	Gm03/LG_N	37.8	37,825,537	99.6	2.9	3.96	−2.20	I	P2
	qRP.3	Gm06/LG_C2	18.0	18,029,734	116.7	10.5	20.10	5.08	I	P1
	​	Gm06/LG_C2	19.6	19,595,695	120.5	12.3	20.85	4.48	II	P1
	​	Gm06/LG_C2	18.0	18,029,734	116.7	9.8	17.70	3.25	IV	P1
	qRP.4	Gm07/LG_M	4.0	3,978,484	18.8	8.1	12.24	−3.95	I	P2
	​	Gm07/LG_M	4.0	3,978,484	18.8	12.1	19.85	−4.31	II	P2
	​	Gm07/LG_M	4.0	3,978,484	18.8	7.4	12.26	−2.64	III	P2
	​	Gm07/LG_M	4.0	3,978,484	18.8	12.2	18.83	−3.35	IV	P2
	qRP.5	Gm17/LG_D2	0.8	0,773,001	1.1	2.5	2.66	1.24	IV	P2
	qRP.6	Gm18/LG_G	8.0	7,955,333	54.4	2.5	4.55	−1.61	IV	P2
	qRP.7	Gm18/LG_G	18.1	18,093,932	67.5	2.5	5.52	−1.80	III	P2
	qRP.8	Gm19/LG_L	43.1	43,084,686	67.7	2.5	4.69	−2.42	I	P2
R8	qR8.1	Gm03/LG_N	37.8	37,825,537	99.6	4.0	2.08	−2.88	I	P2
	qR8.2	Gm05/LG_A1	38.1	38,062,600	106.3	2.9	1.46	2.33	I	P1
	​	Gm05/LG_A1	38.1	38,062,600	106.3	2.7	2.32	2.44	II	P1
	​	Gm05/LG_A1	38.1	38,062,600	106.3	3.1	2.40	2.20	III	P1
	​	Gm05/LG_A1	38.2	38,273,815	107.1	2.8	3.19	2.18	IV	P1
	qR8.3	Gm06/LG_C2	19.6	19,616,246	120.5	45.2	56.12	10.22	III	P1
	​	Gm06/LG_C2	19.7	19,740,115	121.1	52.0	64.48	15.43	I	P1
	​	Gm06/LG_C2	19.7	19,740,115	121.1	51.1	61.38	12.16	II	P1
​	​	Gm06/LG_C2	19.7	19,740,115	121.1	46.1	49.94	11.97	IV	P1
	qR8.4	Gm07/LG_M	4.0	3,978,484	18.8	9.3	7.51	−5.04	I	P2
	​	Gm07/LG_M	4.0	3,978,484	18.8	21.7	16.78	−6.36	II	P2
	​	Gm07/LG_M	4.0	3,978,484	18.8	15.2	12.35	−4.73	III	P2
	​	Gm07/LG_M	4.0	3,978,484	18.8	13.4	13.53	−4.91	IV	P2
	qR8.5	Gm13/LG_F	34.7	34,676,079	94.2	2.6	1.57	−1.98	II	P2
	qR8.6	Gm19/LG_L	45.8	45,757,122	90.9	3.0	1.46	−2.47	I	P2

^a^ Chr/LG, chromosome/linkage group; QPP, QTL, physical position (megabase); QGP, QTL, genetic position (centiMorgan); LOD, logarithm of the odds; PVE, phenotypic variation explained; AD effects, additive effects; Env., environment; I, Stoneville 2017; II, Stoneville 2018; III, Stoneville 2019; IV, BLUP, dataset; P1, DS25-1, P2, DT97-4290.

Altogether eight QTLs were identified for RP (Table 5; Supplementary Figure S2F) on seven different chromosomes (Gm01, Gm03, Gm06, Gm07, Gm17, Gm18, and Gm19), and the LOD score range was relatively narrow (2.5–12.3), compared to other growth-period-related traits (R1 and R8). However, qRP.3 and qRP.4 had much higher LOD scores, 12.3 and 12.1, respectively, and PVE values, 20.85% and 19.85%, respectively, than the other RP QTLs (maximum LOD 2.9 and PVE 5.52%, Table 5). Of these eight, four QTLs, on Gm01 (qRP.1), Gm06 (qRP.3), Gm07 (qRP.4), and Gm18 (qRP.7), were detected consistently, had above-threshold LOD scores, and were considered stable (Supplementary Table S2). The other four QTLs (qRP.2, qRP.5, qRP.6, and qRP.8) were considered minor.

For maturity (R8), six significant QTLs were identified (Table 5; Supplementary Figure S2G), which were distributed on six different chromosomes (Gm03, Gm05, Gm06, Gm07, Gm13, and Gm19) and exhibited a wide range of LOD scores (2.6–52.0). Two major QTLs were identified (Gm06: qR8.3 and Gm07: qR8.4) based on the high LOD scores (52.0 and 21.7, respectively) and PVE (64.48% and 16.58% respectively). Of these six loci, two (Gm06: qR8.3 and Gm07: qR8.4) were detected in multiple environments and thus considered as stable and major QTLs. The remaining four QTLs (qRP.1, qRP.2, qRP.5, and qRP.6) were not detected in multiple environments and were thus considered minor (Table 5).

### Co-localization of QTLs among the traits and candidate gene identification

**FIGURE 2:**
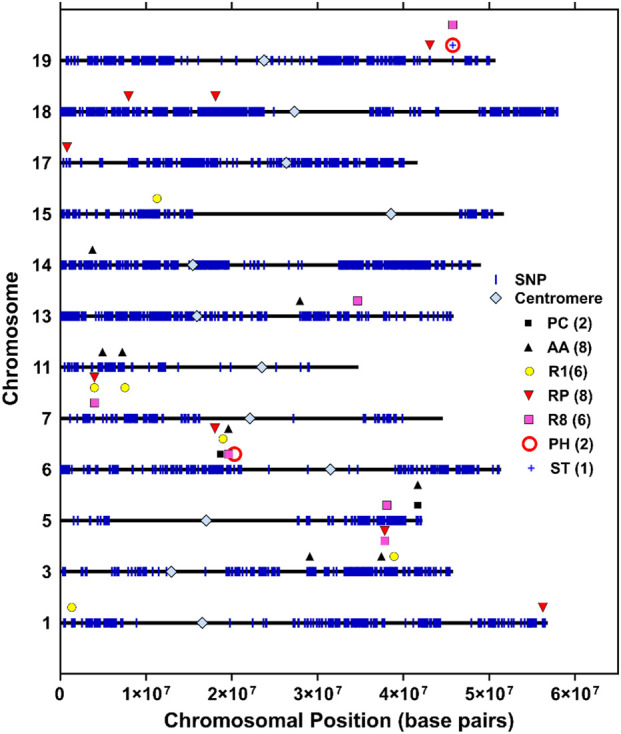
Position of the significant QTLs on the physical map across 12 soybean chromosomes for various traits using the RIL population derived from DS25-1 and DT97-4290. Vertical blue bars represent SNP markers on the map of each chromosome. Different-colored shapes represent QTLs on a specific chromosome for seven traits, namely, pubescence color (PC), accelerated aging (AA%), beginning bloom (R1), reproductive period (RP), maturity (R8), stem termination (ST), and plant height (PH).

We compared QTL results based on their positions and intervals to identify overlapping genomic regions among the seven phenotypic traits (Tables 4, 5; Figure 2). At least four major genomic regions located on chromosomes Gm03, Gm06, Gm07, and Gm19 were identified, exhibiting overlapping loci among multiple phenotypic traits. A genomic region on Gm03 (37.4–38.9 Mb) exhibited co-localized QTLs across the four traits (AA, R1, RP, and R8) between multiple environments. Another genomic region on Gm06 (18.0–20.3 Mb) exhibited co-localization across the six traits (AA, PC, R1, RP, R8, and PH). All these putative QTL loci fell within a region of 2.3 Mb, and most were within a region of 0.9 Mb. Similarly, a genomic region on Gm07 (3.9 Mb) exhibited co-localized QTLs across the growth-period-related traits (R1, RP, and R8). An additional major genomic region on Gm19 (43.1–45.8 Mb) showed co-localized QTLs for PH, ST, and R8 (Tables 4, 5; Figure 2).

The above four genomic regions were considered important, and the regions were analyzed for candidate gene predictions using the Genome Browser at SoyBase (https://www.soybase.org/). We searched for candidate genes within the genomic region approximately 300 kb to the left and right sides of the closest SNP identified for each major QTL (Tables 4, 5) to the corresponding trait and used them for Gene Ontology (GO) analyses. In this analysis, we identified over 362 genes within the selected regions on chromosomes Gm03, Gm06, Gm07, and Gm19 (Supplementary Table S3). Of these, we found key candidate genes near major QTLs identified for different traits. We identified a candidate gene (*Glyma.06G202600*) nearby to our major QTL (qPC.2) located on Gm06 (∼18.6 Mb) for pubescence color that encodes glucan endo-1,3-beta-glucosidase-like protein 2-like. In soybeans, glucan endo-1,3-beta-glucosidases are involved in various biological processes, including plant defense, cell remodeling, cell division, and development (Perrot et al., 2022). *Glyma.06G202600* was 68 kb downstream of a previously detected gene (*Glyma.06G202300*), which encodes cytochrome P450 family proteins (Toda and Yang, 2002; Zhou et al., 2024). Another gene was predicted on chromosome Gm06 (*Glyma.06G207800*), which was previously recognized and cloned as the E1 locus (Bernard, 1972; Xia et al., 2012; Zimmer et al., 2021). Another major genomic region on Gm07 (3.9 Mb) exhibited multiple stable QTLs for R1 (qR1.4), RP (qRP.4), and R8 (qR8.4). Our candidate gene analysis results confirmed the location of the E11 locus (Wang et al., 2019; Zimmer et al., 2021), which was ∼97 kb downstream of our QTL region on Gm07. Additionally, we detected a major genomic region on Gm19 (∼45.8 Mb), which showed multiple co-localized QTLs for PH, ST, and R8. Because we observed a major peak in this region spanning ∼5.0 Mb (43.5–47.2 Mb), we identified several candidate genes within the QTL peak. One of them has been reported as the stem termination *Dt1* gene (*Glyma.19G194300*), which encodes a protein (Terminal Flower 1) in soybean (Liu et al., 2010; Tian et al., 2010).

## Discussion

High seed vigor is a fundamental determinant of seed yield potential, facilitating uniform germination and rapid plant stand establishment. These factors contribute directly to optimal canopy development and resource utilization, thereby maximizing grain yield. The significance of seed vigor is further amplified under abiotic and biotic stress conditions, including sub-optimal temperatures, water-deficit, pathogen pressure, and extreme humidity. Overall, the genetic control of seed vigor in soybeans has not been well studied. The parental lines of this population (DS25-1 and DT97-4290) vary in terms of the characteristics of seed germination, accelerated aging, flowering time, maturity, plant height, stem termination, heat tolerance, mature seed damage tolerance, and seed composition. DS25-1 was derived from a cross between PI 587982A (an unadapted exotic source of abiotic stress tolerance) and DT98-9102, a high-yielding improved germplasm that was publicly released by USDA-ARS but not entered into the USDA germplasm collection (USDA-ARS, 2020). DS25-1 was released by ARS in 2017 (Smith et al., 2017) and has repeatedly demonstrated very high seed germination, even under elevated temperatures (Chebrolu et al., 2016; Gillman et al., 2020; Smith et al., 2025). Because both parental lines of this RIL population showed numerous contrasting breeding-relevant characteristics, our group utilized the population for QTL mapping to understand the genetic control of seed vigor and other important growth-related traits (Tables 1, 4, and 5).

### Trait variations, distributions, heritabilities, and correlations

In this study, a broad range of phenotypic variation was observed between the parental lines and their derived progenies (RILs) for various qualitative and quantitative traits, including AA, PC, R1, RP, R8, PH, and ST (Table 1). This contrasting phenotypic variation across multiple traits allowed us to utilize this population for QTL mapping to identify genomic regions associated with the above traits. The distributions of all the phenotypic traits exhibited continuous variation in a near-normal distribution except for PC and ST, which is expected, given the polygenic nature of AA, R1, RP, R8, and PH (Figure 1). PC and ST are known to be simply inherited. In addition, the RIL population exhibited a broader range of means than their parents, manifesting transgressive segregations for seed vigor and other growth-related traits (AA, R1, RP, R8, and PH), and indicating that comprehensive genetic recombination of alleles had occurred among the progeny of these parental lines (Mackay et al., 2021). The transgressive segregation for corresponding traits can be utilized for trait improvement in soybean breeding programs through pyramiding the desirable allelic combinations contributed from the parents (positive and negative) in the selected progenies.

In this study, most quantitative traits (AA, R1, RP, and R8) exhibited a high broad-sense heritability (0.81–0.96, Table 2), indicating that these traits are heritable and will respond to phenotypic selection. Interestingly, days to maturity (R8, 0.96) exhibited the highest heritability among the traits (AA, R1, RP, and PH) over 3 years of testing, indicating that maturity is a relatively stable trait in this population, wherein perhaps few maturity loci are segregating. Previous studies have also reported high heritabilities for flowering and maturity (Zhang et al., 2015; Liu et al., 2016). In contrast, the moderate heritability for PH (0.52) suggests that this trait is likely influenced by a combination of genetic and environmental factors. One such environmental factor in the current study was planting date, which varied from 10 April 2017 to 22 April 2019. Planting date affects the photoperiod experienced, which affects plant height. In addition, the 2017 experiment was furrow-irrigated, whereas the 2018 and 2019 experiments were rainfed, which may also have affected plant height, although PH was only recorded in 2019.

Relationships between phenotypic traits can play a critical role in breeding programs by suggesting which selection strategy may be most effective for specific trait improvement. Our results demonstrated a significant positive correlation between seed vigor, as measured by AA and major growth-related traits (R1, R8, and PH) across the years (Table 2). Previous studies have reported positive relationships among growth-period traits (Kantolic and Slafer, 2005; Liu et al., 2016) as well as positive correlations with seed yield (Kantolic and Slafer, 2005), which can facilitate simultaneous phenotypic selection for desired trait combinations.

### Candidate genes and consistent QTLs for various traits

Pubescence color (PC) is a relatively well-characterized morphological trait that is used to distinguish soybean genotypes. We identified a major QTL (qPC.2) on Gm06 (∼18.6 Mb) that lies 68 kb downstream of a previously characterized gene (*Glyma.06G202300*) for PC that encodes cytochrome P450 family proteins (Zhou et al., 2024), which are highly expressed in pods and seeds. Previous studies reported that soybean hilum color and pubescence color are pleiotropic and controlled by multiple loci (Bernard and Weiss, 2004), which include a major locus (T/t) previously mapped on Gm06 (Cober and Ablett, 1998) and some minor loci reported on Gm03, Gm04, and Gm05 (Zhou et al., 2024). However, the molecular mechanisms of hilum and pubescence color formation remain unclear. A major QTL (qPC.2) identified for PC overlapped with AA, R1, RP, and R8 (Tables 4, 5). Previous QTL mapping and GWAS studies reported common loci between PC and other traits, such as maturity, growth habit, leaf shape, and cold tolerance (Toda and Yang, 2002; Tsegaw and Zegeye, 2023). Additionally, we detected a minor new locus on Gm05 (∼41.7 Mb, Table 4) for PC, which overlapped with a locus for AA in this study.

QTL analysis identified eight QTLs for AA (Table 4) distributed across six chromosomes (Gm03, Gm05, Gm06, Gm11, Gm13, and Gm14), indicating that AA is polygenic in nature and regulated by multiple genes. Among these eight, two loci (qAA.3 and qAA.4) were detected on Gm05 and Gm06, respectively, in two environments, and both showed positive additive effects. Interestingly, the positive alleles for both these QTLs were contributed from DS25-1, which showed a high AA score (85%) compared to DT97-4290 (45%) and high additive effects (up to 15%) that can be used for stacking these two alleles for improving seed vigor. The location of qAA.3 overlapped with a previously reported QTL on Gm05 (Gillman et al., 2020). It should be noted that DS25-1 likely contains desirable alleles for seed vigor from PI 587982A, which also likely contributed to the improved germination for the parental line (04030-1-4-1-1) used for developing the RIL population tested in the study by Gillman et al. (2020). We identified a candidate gene (*Glyma.06G206400*) close to our major locus (Gm06, qAA.4) that is a DNA-binding transcription factor, which generally plays a critical role in seed development.

### Genomic regions linked to various traits (AA, R1, RP, R8, ST, and PH)

Based on QTL mapping, we identified four genomic regions on Gm03, Gm06, Gm07, and Gm19 that showed overlapping QTLs for multiple traits (AA, PC, R1, RP, R8, and PH; Tables 4, 5). Notably, most of the stable QTLs were detected on Gm06, Gm07, and Gm19 in multiple environments with high LOD scores and were thus considered important for further candidate gene analysis. We found a gene (*Glyma.06G207800*) on chromosome Gm06 that encodes the E1 gene (Bernard, 1972; Xia et al., 2012; Zimmer et al., 2021) in the same interval where we detected QTLs for multiple traits (AA, PC, R1, R8, and PH). It might be due to the confounding effect of the E1 gene. To rule out this possibility, we also performed QTL analysis using maturity data as a cofactor, and we found the same results. Another genomic region on Gm07 (3.9 Mb) showed stable QTLs for three traits (R1, RP, and R8). The closest SNP (Gm07: 3,978,484 bp) linked to the above three QTLs was mapped to ∼97 kb downstream of the reported location of the E11 locus (Wang et al., 2019; Zimmer et al., 2021).

For growth habits, soybeans are generally classified as determinate, semi-determinate, or indeterminate. Determinacy and maturity determine plant architecture, which can play a major role in seed yield potential (Liu et al., 2010; Tian et al., 2010; Ping et al., 2014). Growth habits are genetically governed by two major loci (*Dt1*: Gm19, and *Dt2*: Gm18) mapped on two separate chromosomes (Bernard, 1972; Liu et al., 2010; Ping et al., 2014). The determinate type is regulated by a recessive allele at the *Dt1* locus (Liu et al., 2010), which can also affect flowering and maturity time in soybeans (Bernard, 1972; Zhang et al., 2015; Kong et al., 2018; Ogiso-Tanaka et al., 2019). The semi-indeterminate type is controlled by a dominant epistatic effect of *Dt2* and *Dt1* (Bernard, 1972; Ping et al., 2014). The RIL population used in the current study showed segregation for determinacy. We mapped the *Dt1* (Chr19) locus and confirmed the gene (*Glyma.19G194300*) that encodes a protein (*Terminal Flower 1*) in soybean (Liu et al., 2010; Tian et al., 2010). In this study, the same locus (at ∼45.8 Mb, Gm19) also overlapped with QTLs for PH and ST. This locus accounted for a high amount of the phenotypic variation for PH (28.4%) and ST (65.6%) with negative additive effects for these traits. The desirable alleles for the corresponding traits can be used to select a superior progeny from this population after yield validation. Our findings were corroborated by previous studies (Mansur et al., 1996; Lee et al., 1996; Tian et al., 2022). Other reports have also shown genomic regions that showed common QTLs on the three chromosomes (Gm06, Gm07, and Gm19) for flowering, maturity, and plant height (Kantolic and Slafer, 2005; Fang et al., 2017). Zhang et al. (2015) reported that a major locus on Gm19 (∼45 Mb) exhibited shared associations between multiple traits, including days to flower, days to maturity, and duration of flowering to maturity. Previously, QTL clusters were reported on the overlapping genomic regions associated with the growth-period traits on multiple chromosomes, including Gm06, Gm07, and Gm19 (Liu et al., 2016).

### Other QTLs detected in this study

Several significant QTLs were detected for flowering and maturity on genomic regions different from those of the three overlapping regions described above (Tables 4, 5; Supplementary Table S2). We compared our results with previous GWAS and QTLs studies using SoyBase (https://www.soybase.org/). Two significant QTLs for R1 were identified: one on Gm01 (qR1.1) and one on Gm07 (qR1.4). These coincided with regions found in a previous GWAS study conducted on growth-period traits (Zimmer et al., 2021). However, two additional QTLs were identified for the first time in this study, one on Gm07 (qR1.5) and one on Gm15 (qR1.6). Notably, R1, RP, and R8 are reproductive traits and are correlated in soybean. In this study, QTL qRP.1 and qRP.4 overlapped with full maturity (R8) loci reported previously by Zimmer et al. (2021). Two additional RP QTLs (qRP.2 and qRP.7) detected in this study coincided with those reported from a previous GWAS study (Zhang et al., 2015) for different traits (RP and PH, respectively). In our study, two QTLs, one on Gm17 (qRP.5) and one on Gm18 (qRP.6), were identified for the first time. Maturity (R8) in soybeans is also well-characterized. Although we identified six QTLs linked to R8, only one (qR8.5, Gm13) has not been previously reported. The other five QTLs identified on Gm03, Gm05, Gm06, Gm07, and Gm19 were previously reported (Panthee et al., 2007; Zhang et al., 2015; Zimmer et al., 2021).

## Conclusion

In the present study, an RIL population derived from a cross between DS25-1 and DT97-4290 was phenotypically characterized for various traits, including seed vigor and growth-related characteristics, over three consecutive years. A high-density genetic linkage map comprising 8,445 SNPs was constructed and utilized for composite interval mapping to identify QTLs associated with seven traits: AA, PC, R1, RP, R8, ST, and PH. In total, 33 QTLs were detected across 12 chromosomes (Gm01, Gm03, Gm05, Gm06, Gm07, Gm11, Gm13, Gm14, Gm15, Gm17, Gm18, and Gm19) based on phenotypic datasets from both individual environments and estimated BLUP values. Four genomic regions were found to contain stable QTL in multiple environments, including three chromosomes (Gm06, Gm07, and Gm19) with major QTLs, highlighting the significance of this population for further studies. Within this population, several genetic loci and candidate genes were validated for important traits, including seed vigor, pubescence color, plant height, maturity, and stem termination. Additionally, novel loci associated with seed vigor and growth-related traits were also identified. The discovery of major new QTLs related to seed vigor and growth-related traits provides the basis for developing specific KASP markers for these traits, which can then facilitate marker-assisted selection for the traits in multiple soybean breeding programs.

## Data Availability

The datasets presented in this study can be found in the article/Supplementary Material, further inquiries can be directed to the corresponding author. We request appropriate recognition be made when any data sets are used for producing new publications.
